# Immobilization of a yeast strain isolated from a petrochemical wastewater and effect of phenol on attached cells

**DOI:** 10.1186/1753-6561-8-S4-P216

**Published:** 2014-10-01

**Authors:** Suzana Claudia Silveira Martins, Eduardo Gomes de Almeida Junior, Larissa Maria Cidrão Guedes Fiúza, Claudia Miranda Martins

**Affiliations:** 1 Department of Biology, Pici, Bl. 909, 60455-760, Federal University of Ceara, Fortaleza, Brazil

## Background

Cell immobilization is a strategy to enhance the efficiency of biorremediation processes [1]. Polyurethane foams have gained relevance for their resistance to microbial attack and cost effectiveness. Although the phenotypic diversity and lower generation time of bacteria make this group the most studied, yeasts are also able to adhere and grow on inert surfaces. While free yeast cells have been studied for use in bioremediation, only in the past two decades have studies of immobilized yeast cells been reported [2]. Among the organic environmental pollutants, phenol stands out for its recalcitrance and toxicity. Therefore, the aims of this work were: (i) to evaluate the potential for immobilization of a yeast strain from a petrochemical wastewater in Brazil, (ii) to test flexible polyurethane foam as immobilizing agent for this strain and (iii) to compare the effect of phenol on immobilized and free cells.

## Methods

The *Candida rugosa *yeast strain used in this work was isolated from oil refinery wastewater polluted by phenol. The cell surface hydrophobicity was determined by the replica method [3]. Adhesion to of xylene was evaluated according to the hidrophobicity method [4]. Qualitative production of biofilm was tested by cultivation on Congo red agar (CRA) and microbial adhesion in glass tubes (MAG) [5]. The cell suspension of *C. rugosa *was standardized to optical density of 0.5 at 600 nm, corresponding to an inoculum of 10^8^-10^9 ^CFU mL^-1^. The strain was cultived on sterilized polyurethane foam matrices. The number of colony-forming units per square centimeter (CFU cm^-2^) of polyurethane was determined using the spread plate method. Free and immobilzed cells were cultured in a synthetic mineral medium containing different phenol concentrations. Tolerance to phenol was determined by daily measurements of CFU cm^-2^.

## Results and conclusions

The index of partition and translucent violet areas classified the strain as moderately hydrophobic. Dark red colonies on CRA plates and wall glass tubes stained red indicated that the *C. rugosa *strain is a biofilm producer. The growth curve of *C. rugosa *showed three phases: (1) lag phase, from 0 to 2 h, during which the number of adhered cells was 4.3 Log CFU cm^-2^; (2) logarithmic phase, which lasted from 2 to 10 h, when the cell count increased from 4.3 Log CFU cm^-2 ^to 6.4 Log CFU cm^-2^; and (3) stationary phase, which lasted from 10 to 24 h, when the cell concentration was 6.4 Log CFU cm^-2^. The concentration achieved after 24 h, around 2.5 × 10^6 ^CFU cm^-2^, is suggestive of biofilm formation. Concentrations of 62.5 mg L^-1^, 125 mg L^-1^and 250 mg L^-1 ^of phenol had a lethal effect on the cells in suspension. The increase of the number of cells at 62.5 mg L^-1 ^and 125 mg L^-1 ^of phenol indicated the protective effect of the cell immobilization. *C. rugosa *from petrochemical wastewater presented hydrophobicity and biofilm production, indicating the ability to adhere on solid surfaces. Immobilization of this strain on polyurethane foam might be a useful strategy to improve bioremediation and for treatment of phenol-polluted industrial and petrochemical wastewater.
